# Different Functions of *IbRAP2.4*, a Drought-Responsive AP2/ERF Transcription Factor, in Regulating Root Development Between *Arabidopsis* and Sweetpotato

**DOI:** 10.3389/fpls.2022.820450

**Published:** 2022-01-26

**Authors:** Xiaofeng Bian, Ho Soo Kim, Sang-Soo Kwak, Qian Zhang, Shuai Liu, Peiyong Ma, Zhaodong Jia, Yizhi Xie, Peng Zhang, Yang Yu

**Affiliations:** ^1^Institute of Food Crops, Provincial Key Laboratory of Agrobiology, Jiangsu Academy of Agricultural Sciences, Nanjing, China; ^2^Plant Systems Engineering Research Center, Korea Research Institute of Bioscience and Biotechnology (KRIBB), Daejeon, South Korea; ^3^National Key Laboratory of Plant Molecular Genetics, CAS Center for Excellence in Molecular Plant Sciences, Institute of Plant Physiology and Ecology, Chinese Academy of Sciences, Shanghai, China

**Keywords:** sweetpotato (*Ipomoea batatas* (L.) Lam), *Arabidopsis*, AP2/ERF, lateral root, storage root, DRE element, GCC-box

## Abstract

Plant root systems are essential for the uptake of water and nutrients from soil and are positively correlated to yield in many crops including the sweetpotato, *Ipomoea batatas* (L.) Lam. Here, we isolated and functionally characterized *IbRAP2.4*, a novel nuclear-localized gene encoding the AP2/ERF transcription factor, from sweetpotato. *IbRAP2.4* was responsive to NaCl, PEG8000, ethylene, and Indole 3-acetic acid treatments. As revealed by electrophoretic mobility shift assay and dual luciferase assay, IbRAP2.4 could bind to both DRE and GCC-box elements and acted as a transcription activator. *IbRAP2.4* overexpression significantly promoted lateral root formation and enhanced the drought tolerance in *Arabidopsis thaliana*, while it inhibited storage root formation in transgenic sweetpotato by comprehensively upregulating lignin biosynthesis pathway genes. Results suggested that *IbRAP2.4* may be a useful potential target for further molecular breeding of high yielding sweetpotato.

## Introduction

Plants sense a wide array of stressors in their environment, including dehydration, which adversely affect their growth and yields. Plant roots uptake water and nutrients, provide anchorage, and monitor environmental conditions in the soil. To ensure plants’ survival in changing environments, root systems develop various adaptive traits governed through a complicated mechanism. Therefore, elucidating the molecular mechanisms underlying root architecture can contribute to the improvement of crop yields and stress tolerance.

Plant root systems develop from the root apical meristem initiated during embryogenesis and possibly from multiple non-root-borne adventitious root apical meristems (Motte et al., 2019). Plant root systems exhibit a wide variety of morphologies depending on the species. The model plant, *Arabidopsis thaliana*, has a tap-root system consisting of a central primary root and lateral roots (LRs), whereas several tuber and corm crops such as sweetpotato [*Ipomoea batatas* (L.) Lam.], develop an adventitious root system that emerges from diverse non-root organs after the embryonic stage (Belehu et al., 2004; Guan et al., 2019; Wei et al., 2019). The sweetpotato root system includes “thick” pigmented storage roots (SRs), “thick” pigmented pencil roots (PRs), and white fibrous roots (FRs) (Wilson and Lowe, 1973; Belehu et al., 2004).

The formation of a plant root system is a strictly controlled developmental process. Accumulating investigations indicated that phytohormones, such as auxins and ethylene, and some key factors involved in hormone signaling, play an essential role in the regulation of root development (Liu et al., 2016; Qin and Huang, 2018; Yoon et al., 2020). The AP2/ERF transcriptional factors, known to play very important roles in hormonal regulation, have been reported to be involved in root architecture regulation in a diverse range of plants. For instance, the degradation of ERF13 induced by auxins could positively regulate LR development in *Arabidopsis* (Lv et al., 2021). *OsERF2*, a transcription factor required for the interplay between ethylene and ABA, negatively regulates primary root growth in rice (Xiao et al., 2016). Moreover, both *OsERF3* and *OsCRL5* could positively regulate the rice response regulators (RRs) of cytokinin signaling during crown root initiation (Kitomi et al., 2011; Zhao et al., 2015). The AP2 DNA-binding domain containing transcription factor family is large group of plant-specific proteins which can be classified into four major subfamilies: AP2 (APETALA2), ERF (Ethylene-Responsive-Element-Binding protein), DREB (Dehydration Responsive Element-Binding), and RAV (Related to ABI3/VP) (Nakano et al., 2006). AP2/ERF transcription factors have also been reported involving in plant (a)biotic stress responses by regulating downstream genes expression *via cis-*acting elements, such as dehydration responsive elements (DRE)/C-repeat element (CRT) and/or GCC box (Lee et al., 2015; Xie et al., 2019). Recently, several genes belonging to the AP2/ERF transcription factor family have been isolated from sweetpotato (Kim Y. H. et al., 2012; Li et al., 2019; Yu et al., 2020). However, none is reported to regulate root development.

Sweetpotato is a food crop cultivated in all tropical and subtropical regions, particularly in Asia, Africa, and the Pacific (El Sheikha and Ray, 2017). SRs constitute the most economically important agronomic trait in sweetpotato production; therefore, understanding the mechanisms controlling root development is crucial for high yields breeding of sweetpotato. To reveal how AP2/ERF transcription factors affected sweetpotato root development, we isolated and functionally characterized a novel nuclear-localized AP2/ERF transcription factor, *IbRAP2.4*, in sweetpotato. IbRAP2.4 could directly bind with the DRE and GCC-box elements where it acted as a transcription activator. *IbRAP2.4* overexpression significantly promoted LR formation and enhanced drought tolerance in *Arabidopsis thaliana*, whereas it simultaneously and substantially inhibited the formation of SRs but induced PRs in sweetpotato. The expression levels of *cinnamoyl*-*CoA reductase* (*CCR*), *caffeic acid*/*5*-*hydroxyferulic acid O*-*methyltransferase* (*COMT*), and *caffeoyl*-*CoA O*-*methyltransferase* (*CCoAOMT*) genes involved with lignin biosynthesis were increased in *IbRAP2.4*-overexpressing transgenic sweetpotato. Functional analysis of *IbRAP2.4* regarding the regulation of root architecture provides a framework for the future breeding of high yielding sweetpotato.

## Materials and Methods

### Plants Materials and Growth Conditions

Sweetpotato cultivar Sushu 16 (bred by Jiangsu Academy of Agricultural Sciences) and Xushu 29 (bred by Jiangsu Xuzhou Sweetpotato Research Center), and *Arabidopsis thaliana* ecotype Colombia-o (Col-o) were used in this study. Xushu 29 and Col-o plants were used as the wild-type (WT) to generate transgenic lines. Sweetpotato plants were propagated by cuttings (tips: top 10 cm) and grown in a growth chamber under a 16 h light/8 h dark photoperiod at 25°C. The seeds of *Arabidopsis* were surface sterilized, sown on 1/2 Murashige and Skoog (MS) medium, and then grown in a growth chamber under a 16 h light/8 h dark photoperiod at 22°C. Untransformed and transgenic sweetpotato plants were transplanted into the field in mid-May for evaluation of the phenotype in Nanjing, Jiangsu. For gene expression pattern analysis, sweetpotato seedlings were cultured in 1/2 Hoagland nutrient solution for 10 d and subsequently subjected to stress treatments by supplementing with 150 mM NaCl (salt stress), 20% polyethylene glycol (PEG) 8,000 (drought stress), 100 μM ethylene, and 100 μM indole-3-acetic acid (IAA), respectively. The fourth fully expanded leaf from the top was sampled at 0, 3, 6, 12, 24, 48 h after treatment. Plant tissues (leaves, stems, FRs, PRs, and SRs) collected from the 10-week-old sweetpotato plants under normal growth conditions were used for tissue-specific expression analysis. For drought stress treatment of *Arabidopsis*, after being sown in pots and regularly watered for 2 weeks, transgenic plants and WT were subjected to withholding of watering for 7 days followed by 1 day of recommenced watering.

### Isolation and Analysis of IbRAP2.4

The *IbRAP2.4* gene was predicted by the Sushu 16 transcriptome database, and isolated from the cDNAs of Sushu 16 by using sequence-specific primers (Supplementary Table 1). The PCR products were then fused into the pEASY-Blunt cloning vector (TransGen, Beijing, China) for sequencing confirmed with five independent clones.

The conserved domain of the IbRAP2.4 protein was scanned by CD-search.^1^ Homologous sequences of IbRAP2.4 were identified using the BLASTp search program of the National Center for Biotechnology Information.^2^ Molecular phylogenetic analysis was constructed using the UPGMA method and MEGA 6.^3^ Sequence alignment was conducted with BioEdit.^4^ A 2.0 kb-length promoter region of *IbRAP2.4* exported from the sweetpotato genome database^5^ were analyzed by the PlantCARE.^6^

### Transcriptional Activation Assay of IbRAP2.4

The full-length *IbRAP2.4* coding sequence (CDS) was inserted into the pGBKT7 vector to produce the fusion construct pGBKT7-IbRAP2.4 (Primer pair RAPY-F/R, Supplementary Table 1). The generated plasmid pGBKT7-IbRAP2.4 and the empty pGBKT7 vector (negative control) were transformed into the yeast (*Saccharomyces cerevisiae*) strain AH109 separately. Yeast cells grown on SD/-Trp and SD/-Trp/-His/-Ade plates were incubated at 30°C for 3–5 days.

### Subcellular Localization

Subcellular localization analysis of IbRAP2.4 was determined in 3-week-old tobacco (*Nicotiana benthamiana*) leaves and conducted as described previously (Waadt and Kudla, 2008). The coding sequence of *IbRAP2.4* was amplified and constructed into the transient expression vector pCAMBIA1305-GFP (primer pair RAPG-F/R, Supplementary Table 1). The construct was transformed into *Agrobacterium tumefaciens* strain GV3101 and then used to infiltrate *N. benthamiana* leaves. The infiltrated *N. benthamiana* were maintained in a growth chamber under a 16 h light/8 h dark photoperiod at 25°C for 2 days. Samples with 4,′6-diamidino-2-phenylindole (DAPI) staining were collected for detection of fluorescent signal with a Leica TCS SP5 confocal laser scanning microscope.

### Gene Expression Level Analysis

Total RNA of samples was isolated by using RNAprep Pure Plant kit (Polysaccharides and Polyphenolics-rich) (Tiangen, Beijing, China) and then used to synthesize the first-strand cDNA with FastKing gDNA Dispelling RT SuperMix kit (Tiangen, Beijing, China). Primer pairs specific to tested genes were designed using the GenScript online tool^7^ and then used to quantify the expression level by quantitative real-time PCR (qRT-PCR) amplification (Supplementary Table 1). qRT-PCR was performed using a SYBR^®^ Premix Ex Taq™ kit (TaKaRa) on an ABI prism 7,900 Real-Time PCR System. The sweetpotato *Tublin* gene was used as a reference and relative changes of gene expression was analyzed by the 2^–ΔΔCT^ method (Livak and Schmittgen, 2001).

### Vector Construction and Plant Transformation

The full length sequence of *IbRAP2.4* was cloned into the plant expression vector pCAMBIA1305 behind the *CaMV35S* promoter to generate *CaMV35S*:IbRAP2.4 construct (Primer pair ORAP-F/R, Supplementary Table 1). *CaMV35S*:IbRAP2.4 plasmid was transferred into *Agrobacterium tumefaciens* EHA105 and introduced into Col-o and Xushu 29, respectively, as previously described (Clough and Bent, 1998; Kim S. H. et al., 2012). For *Arabidopsis*, the first generation (T_0_) seeds were germinated on 1/2 MS medium with 40 mg L^–1^ hygromycin for screening. For sweetpotato, the transformed calli were selected on MS medium containing 400 mg L^–1^ cefotaxime and 25 mg L^–1^ hygromycin. All transgenic lines were confirmed by PCR amplification of hygromycin and qRT-PCR with specific primers (Primer pair hyg-F/R, Supplementary Table 1).

### Purification of GST-IbRAP2.4 Fusion Protein and Electrophoretic Mobility Shift Assay

The GST:IbRAP2.4 plasmids was transformed to *E. coli* strain BL21 (DE3) and the recombinant protein was purified by affinity chromatography using a Glutathione Sepharose 4B column (GE Healthcare, Uppsala, Sweden) according to the manufacturer’s protocols. The DRE (5′-TTGATACTACCGACATGAGTTGATACTACCGACATGAGTT -3′) and GCC-box (5′-TTCATAAGAGCCGCCACTCATAAGAG CCGCCACT-3′) sequences used as probes were according to the findings of Lin et al. (2008). To prepare probes, oligonucleotide sets were annealed by boiling for 5 min and then labeled with [γ-^32^P]-ATP by adding T4 Polynucleotide Kinase (Promega, Madison, WI, United States). A 30 μL binding reactions were performed by the addition of labeled probe (0.5 μg) and purified GST-IbRAP2.4 or GST protein (10 μg) into the binding buffer (200 mM HEPES, 50 mM KCl, 5 mM DTT, 1 mM EDTA, and 20 pmol of poly dI-dC) and then allowed to proceed at room temperature for 30 min. Afterward, the sample was electrophoresed on an 8% native polyacrylamide gel and visualized by autoradiography. Competition experiments were carried out by incubating increasing amounts of unlabeled competitor probe with purified GST-IbRAP2.4 fusion protein before the addition of labeled probes.

### Assay of Luciferase Activity and Chlorophyll Content

The 4 × DRE and 3 × GCC fragments were inserted to pGreen 0800-Luc vector, respectively. The reporter (4 × DRE-Luc/3 × GCC-Luc) and effector (35S:IbRAP2.4) (or empty vector) were co-transformed into *Arabidopsis* protoplasts via a PEG transformation method, performed as previously described (Iwata et al., 2011). Luciferase activities were measured with the Dual-Luciferase Reporter Assay System.^8^ To measure the chlorophyll content in transgenic *Arabidopsis*, the fourth fully expanded leaves from the top of transgenic lines and WT plants were collected for detection using a portable chlorophyll meter (SPAD-502, Konica Minolta, Japan).

### Statistical Analysis

Three biological replicates were performed for all experiments. Difference analysis of data presented as the mean ± standard deviation (SD) was performed with Student’s t-test (two-tailed analysis) using SPSS, version 20.0 (IBM Corporation, Armonk, NY, United States). Significance levels at *P* < 0.05, and *P* < 0.01 are indicated by * and **, respectively.

## Results

### Isolation and Characterization of IbRAP2.4

**FIGURE 1:**
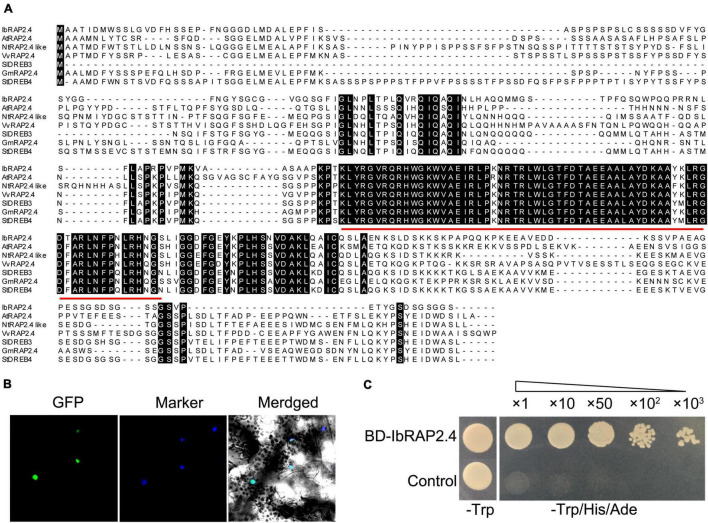
Characterization of IbRAP2.4. **(A)** Amino acid sequence alignment of IbRAP2.4 and AtRAP2.4. Conserved AP2 domain is emphasized with a red line. **(B)** Subcellular localization of IbRAP2.4. **(C)** Transactivation assay of the IbRAP2.4 protein. Triangles indicate increasing dilutions (×1, ×10, ×50, ×100, and ×1000).

The full-length CDS of a candidate gene predicted encoding an ERF transcription factor was obtained from the Sushu 16 transcriptome database. The gene showed a complete open reading frame of 885 bp (GenBank: KX255653.1), encoding a protein of 294 amino acids (GenBank: ARS72979.1) with a molecular weight of 30.83 kDa. Analysis of the deduced amino acid residues revealed that the protein contained a typical AP2 domain (135–197) (Figure 1A). As the protein showed the highest similarity with AtRAP2.4 from *Arabidopsis* (*At1g78080*), which was classified into the DREB subfamily (Lin et al., 2008), we named it IbRAP2.4 (Figure 1A). A phylogenetic tree was constructed with the amino acid sequences of IbRAP2.4 and DREB proteins from other plants. The results revealed that IbRAP2.4 was closely related to the homologs of grape (*Vitis vinifera*), soybean (*Glycine max*), and *Coffea arabica* (Supplementary Figure 1).

To determine the subcellular localization of IbRAP2.4, the coding region of *IbRAP2.4* was fused with GFP and transiently expressed in tobacco (*N. benthamiana*) leaves. Confocal microscopic analysis showed that the fusion protein IbRAP2.4-GFP was specifically localized to the nuclei, suggesting that IbRAP.4 is a nuclear-localized protein (Figure 1B). Yeast transcriptional activation assay of IbRAP2.4 revealed that both the control and yeast cells harboring BD-IbRAP2.4 grew well on SD/-Trp medium. While yeast cells harboring BD-IbRAP2.4 grew well on SD/-Trp/-His/-Ade medium, the control did not (Figure 1C). These results indicated that IbRAP2.4 had transcriptional activity in yeast.

### Expression Patterns of IbRAP2.4

**FIGURE 2:**
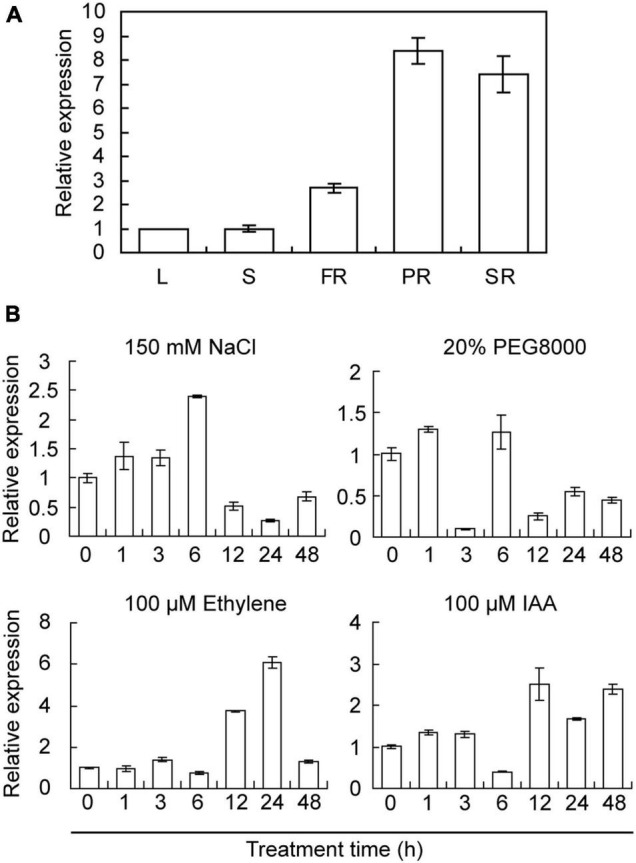
Expression patterns of *IbRAP2.4*. **(A)** Tissue-specific expression pattern of *IbRAP2.4*. L, leaf; S, stem; FR, fibrous root; PR, pencil root; SR, storage root. **(B)** Expression pattern of *IbRAP2.4* in responses to abiotic stresses and hormones. Data represent mean ± standard deviation (SD) of three biological replicates.

The expression profiles of *IbRAP2.4* in various tissues of sweetpotato were analyzed by qRT-PCR. *IbRAP2.4* was constitutively expressed in all tested tissues, with higher expression in the pigment roots, SRs, and FRs, indicating its critical function in sweetpotato roots (Figure 2A). Characterization of *cis*-acting elements in the promoter region of *IbRAP2.4* revealed that some stress- and phytohormone-responsive sites were involved, such as AuxRR-core, ERE, ABRE, and TC-rich repeats (Supplementary Figure 2). To further explore its potential function in response to abiotic stresses and hormones, the expression of *IbRAP2.4* was detected in Sushu 16 seedlings under different treatments. As shown in Figure 2B, the expression of *IbRAP2.4* was upregulated by NaCl, beginning to increase at 1 h, peaking at 6 h, and then decreasing. Under PEG8000 stress, the expression of *IbRAP2.4* was slightly induced at only 1 and 6 h, but was significantly decreased at 3 h and after 12 h. When treated with ethylene, transcription of *IbRAP2.4* was upregulated at 12 h, reached its maximum at 24 h and decreased at 48 h. For IAA treatment, after a slight increment in the first 3 h, the expression of *IbRAP2.4* decreased immediately at 6 h but was induced dramatically over the following 12 h. Taken together, these results suggest that *IbRAP2.4* is induced by salinity, dehydration, ethylene, and IAA.

### IbRAP2.4 Binds to the Dehydration Responsive Elements and GCC-Box Elements and Acts as a Transcriptional Activator

**FIGURE 3:**
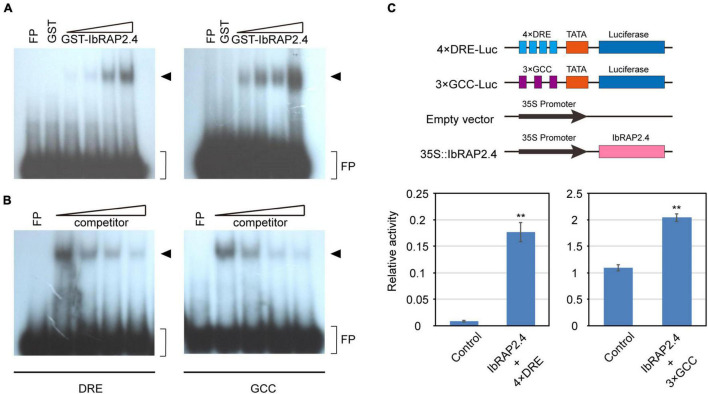
Binding of IbRAP2.4 to the DRE element and GCC-box. **(A)** Dosage-dependent binding of GST-IbRAP2.4 to the DRE element and GCC-box. Triangles indicate increasing amounts of GST-IbRAP2.4 protein (0.5, 1.0, 2.0, and 4 μg) used for DNA-binding analysis. The DNA-protein-binding complexes are indicated with an arrowhead. FP, free probe. **(B)** Competition analysis of unlabeled probe. Triangles indicate increasing amounts of unlabeled probe. The DNA-protein-binding complexes are indicated with an arrowhead. FP, free probe. **(C)** IbRAP2.4 activates the 4 × DRE-Luc reporter and the 3 × GCC-Luc reporter gene expression in *Arabidopsis* protoplasts. Bars represent standard deviations of triplicate experiments. Asterisks indicate significant differences between control and effector at **P < 0.01.

It has been reported that amounts of AP2-domain containing proteins can bind to the dehydration responsive element (DRE, core sequence TACCGACAT) or the ethylene-responsive GCC-box (core sequence AGCCGCC) (Lin et al., 2008). To directly test the DNA-binding activity of IbRAP2.4, we performed an electrophoretic mobility shift assay (EMSA) using recombinant GST-IbRAP2.4 fusion protein and labeled DRE or GCC-box element. The results showed that GST-IbRAP2.4 fusion protein, but not GST alone, can bind with both the DRE and GCC-box in a dosage-dependent manner (Figure 3A). Excess amounts of unlabeled probes can effectively reduce or abolish the binding of GST-IbRAP2.4 to the labeled DRE or GCC-box element (Figure 3B). These results indicate that IbRAP2.4 can specifically bind to both the DRE and GCC-box element *in vitro*.

To determine whether IbRAP2.4 acts as a transcriptional activator or repressor, its transcriptional activity was examined by dual luciferase assay (Dual-Luc) in *Arabidopsis thaliana* protoplasts. The reporter plasmid contained the luciferase (*LUC*) gene fused to four copies of the DRE element or three copies of the GCC-box element, and the effector plasmid contained full-length CDS of *IbRAP2.4* with double copies of the 35S promoter (Figure 3C). The luciferase activity showed a more than two-fold increment in the presence of IbRAP.4 protein, compared to that of the control, indicating strong activator activity of *IbRAP2.4* in transcriptional regulation of DRE-containing and GCC-box-containing gene expression in plant cells.

### Overexpression of IbRAP2.4 Promoted Lateral Root Formation and Enhanced Drought Tolerance in Transgenic *Arabidopsis thaliana*

**FIGURE 4:**
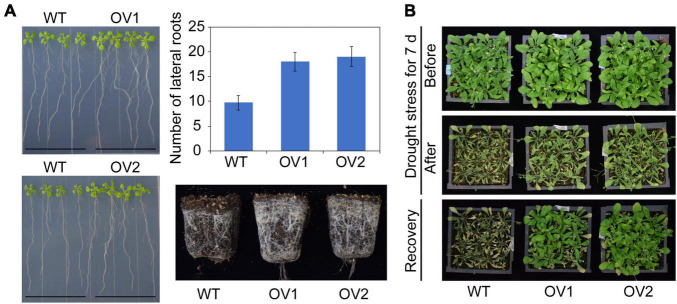
Promoted lateral root (LR) formation **(A)** and enhanced drought tolerance **(B)** in transgenic *Arabidopsis* plants overexpressing *IbRAP2.4* (OV1 and OV2). Data represent mean ± SD of three biological replicates.

To further characterize the function of *IbRAP2.4* in plants, we firstly generated transgenic *Arabidopsis thaliana* that overexpressed *IbRAP2.4*. Two independent T3 homozygous lines (OV1 and OV2) of *Arabidopsis* overexpressing *IbRAP2.4* (data not shown) with obvious phenotypes were selected as representatives. Compared with WT, the LR formation of *Arabidopsis* transgenic plants was affected by *IbRAP2.4* overexpression (Figure 4A). A notably promoted LR formation was observed in OV lines at day 12 after sowing, and the post-harvest total root production in OV lines was obviously increased owing to accelerated LR growth. In addition, OV plants displayed enlarged rosette leaves, both in length and width. The biomass of *IbRAP2.4* overexpressed plants was higher than that of the control. In contrast, overexpression of *IbRAP2.4* caused a reduction in chlorophyll content (Supplementary Figure 3).

To reveal whether *IbRAP2.4* contributed to the tolerance of drought stress, both the transgenic lines and WT grown in pots for 2 weeks were treated with drought. No obvious difference was observed between WT and OVs under normal conditions, whereas the WT plants exhibited increased sensitivity to drought stress compared with the overexpressed-*IbRAP2.4* transgenic lines (Figure 4B). After re-watering for 1 day, plants of transgenic *Arabidopsis* lines OV1 and OV2 only displayed slight wilting and successfully recovered, while most of the WT nearly died with their leaves turned yellow and were shrunken in size. All these results illustrate that overexpressed *IbRAP2.4* promoted LR formation and enhanced drought tolerance in *Arabidopsis*.

### Overexpression of IbRAP2.4 Affected Root Formation in Sweetpotato

**FIGURE 5:**
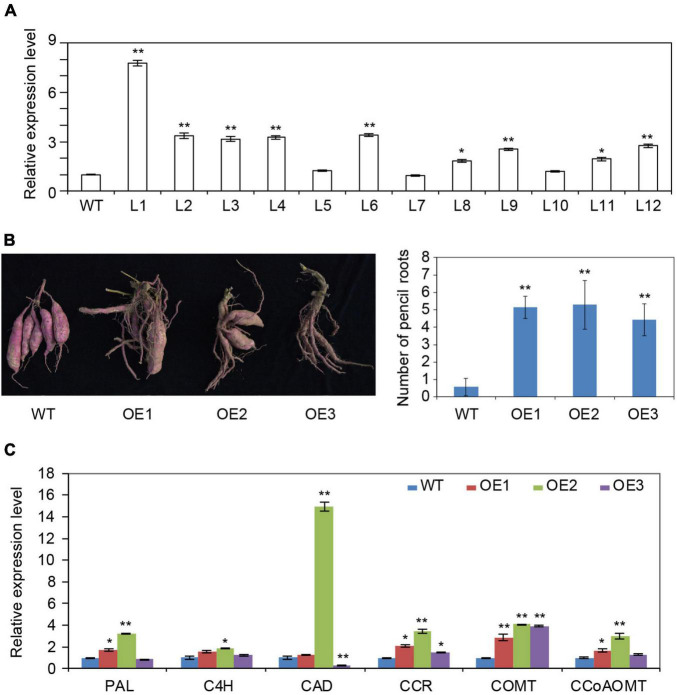
Overexpression of *IbRAP2.4* in sweetpotato. **(A)** qRT-PCR analysis of sweetpotato transgenic lines overexpressing *IbRAP2.4* (L1 to L12). **(B)** Reduced number of storage roots (SRs) but increased number of pencil roots (PRs) produced by representative transgenic lines (OE1 to OE3). **(C)** Expression of genes involved in lignin biosynthesis. Data represent mean ± SD of three biological replicates. The asterisks represent significant differences from the WT at *P < 0.05 or **P < 0.01.

To confirm the role of *IbRAP2.4* in sweetpotato, it was transformed into Xushu 29, a variety known to be suitable for transformation, to generate overexpression lines. A total of 12 transgenic lines were obtained and confirmed by PCR (data not shown) and qRT-PCR (Figure 5A). Three transgenic lines (L1 to L3) exhibiting high *IbRAP.4* expression were selected as representatives (OE1 to OE3) for further characterization. The transgenic lines and WT plants showed no differences in response to drought stress (data not shown). However, after the plants were harvested from the field, obvious phenotypic changes were observed in the mature storage roots in *IbRAP2.4* overexpression transgenic lines compared to the WT (Figure 5B and Supplementary Figure 4). Under field conditions, WT produced 3–5 storage roots per plant, whereas the number of SRs in OE lines was notably reduced. In contrast, more PRs were produced by OE lines, ranging from 4.43 (in L3) to 5.29 (in L2); this was far superior to the WT (0.57).

As PRs are a type of lignified thick root, it was speculated that lignin biosynthesis in transgenic sweetpotato might be impacted by overexpressing *IbRAP2.4*. The expression of key enzyme genes involved in lignin biosynthesis, including *phenylalanine ammonia*-*lyase* (*PAL*), *cinnamate 4*-*hydroxylase* (*C4H*), *cinnamyl alcohol dehydrogenase* (*CAD*), *cinnamoyl*-*CoA reductase* (*CCR*), *caffeic acid*/*5*-*hydroxyferulic acid O*-*methyltransferase* (*COMT*), and *caffeoyl*-*CoA O*-*methyltransferase* (*CCoAOMT*), was analyzed by qRT-PCR using 5-week-old seedlings (Figure 5C). Compared to the WT, the three OE lines showed 2.88- to 4.12-fold changes in the expression of *COMT*. A slight up-regulation with 1.53- to 3.43-fold changes was also observed in the expression of *CCR* in transgenic lines. These results indicate that overexpression of *IbRAP2.4* inhibited SR formation by activating the expression of genes involved in the lignin biosynthesis pathway.

## Discussion

We identified the *IbRAP2.4* gene as a novel regulator of root development. The IbRAP2.4 protein shares high similarity with *Arabidopsis* RAP2.4 which belongs to the DREB subfamily of AP2/ERF family (Figure 1). Many members of the AP2/ERF family are known to bind to either the DRE element or the ethylene-responsive GCC-box, while others bind to both of the two *cis*-elements (Phukan et al., 2017; Xie et al., 2019). In accordance with *Arabidopsis* RAP2.4 (Lin et al., 2008), IbRAP2.4 can bind to both the DRE and GCC-box *cis*-elements, and acts as a transcription activator (Figure 3). DREBs have been extensively examined under abiotic stress, where they respond to and positively regulated drought, cold, salt, and heat tolerance by regulating stress-responsive genes (Xie et al., 2019). Drought- and salt- inducible *Arabidopsis RAP2.4* is reported to regulate multiple developmental processes and drought stress tolerance (Lin et al., 2008; Yang et al., 2020). In this study, *IbRAP2.4* expression was highly detected in sweetpotato roots (Figure 2A), and induced by treatments of salinity, dehydration, ethylene, and IAA (Figure 2B). In addition to the enhanced drought tolerance in *IbRAP2.4* OVs, overexpression of *IbRAP2.4* in *Arabidopsis* and sweetpotato displayed promoted LRs formation and increased PRs respectively (Figures 4, 5), illustrating the regulatory role of *IbRAP2.4* in plant root development.

Lateral roots in *IbRAP2.4*-overexpression *Arabidopsis* lines exhibited promoted formation. The whole process of LR development in *Arabidopsis* has been well established and auxins play essential roles in each developmental phase (Du and Scheres, 2018). AP2/ERF transcription factors were found to be involved in auxin-regulated LR development. The auxin-regulated PUCHI gene, encoding an AP2/ERF transcription factor, is required for a coordinated pattern of cell division during LR formation in *Arabidopsis* (Hirota et al., 2007). ERF109-mediated cross-talk of JA signaling and auxins regulate LR formation by directly activating auxin biosynthesis genes (Cai et al., 2014). Recently, Lv et al. (2021) reported that *ERF13* acts as a negative regulator of LR development that is degraded upon auxin perception through an MPK14-dependent pathway. Although the expression of *IbRAP2.4* in sweetpotato was induced by the active auxin IAA (Figure 2B), further confirmation is needed as to whether *IbRAP2.4-*controled LR development depends on an auxin-regulated pathway.

*Arabidopsis* lines expressing *CaMV35S*:*IbRAP2.4* showed improved drought tolerance as compared to WT (Figure 4B). Previous studies have implicated several *Arabidopsis* AP2/ERF family genes in drought stress tolerance but with different mechanisms. For instance, transgenic alfalfa (*Medicago sativa*) overexpressing WXP1 with increased cuticular wax accumulation on leaf surfaces shows enhanced drought tolerance (Zhang et al., 2005, 2007). Whereas overexpression of *OsERF71* changes the root structure of plants through modification of its cell wall, thereby enhancing rice tolerance to drought stress (Lee et al., 2016). A direct correlation between root architecture and resistance to water deficit has been shown in many crop plants. Root-specific overexpression of three *OsNACs* (*OsNAC5*, *OsNAC59*, and *OsNAC510*) notably enlarges roots, which in turn enhances drought tolerance (Jeong et al., 2010, 2013; Redillas et al., 2012). In addition, overexpression of DEEPER ROOTING1 confers a drought avoidance capacity by altering rice root architecture (Uga et al., 2013). LRs increase the contact area of roots with soil particles and thereby aid water and nutrient absorption from the soil (Ahmed et al., 2016). A previous study also elucidates that higher LR density can potentially enhance drought tolerance for plants (Placido et al., 2020). Therefore, we speculated that the enhanced drought tolerance in overexpressed *IbRAP2.4* transgenic *Arabidopsis* may be a result of its promoted LR development. Furthermore, *IbRAP2.4*-overexpression sweetpotato lines did not show a significant phenotype for drought tolerance, which may be explained by the different regulatory mechanisms of root system formation between *Arabidopsis* and sweetpotato.

Sweetpotato yield and quality are dependent upon SR development during plant growth. Therefore, much of the research on sweetpotato focuses on the mechanisms underlying SR formation and development. The sweetpotato plant initially produces colorless FRs, with some of these subsequently acquiring pigmentation and undergoing “thickening” growth to form PRs that ultimately develop into SRs. Recently, a number of genes related to SR formation have been identified in sweetpotato, such as: *SRD1*, which is essential for the initiation and development of tuberous roots by influencing auxin synthesis (Noh et al., 2010); expansion gene *IbEXP1*, which affects the weight and number of SRs (Noh et al., 2013); and *IbNAC083*, a core regulator of SR swelling initiation (He et al., 2021). Results of overexpressed *IbRAP2.4* transgenic sweetpotato suggested a role for the AP2/ERF transcription factor in SR development (Figure 5 and Supplementary Figure 4). Lignification of the middle column inhibits the conversion of adventitious roots to SRs (Belehu et al., 2004), and transcriptomic analysis validated the down-regulation of lignin biosynthesis and up-regulation of starch biosynthesis related genes at an early stage of SR formation (Firon et al., 2013). Consistent with these conclusions, inhibited SR formation in *IbRAP2.4-*overexpression sweetpotato lines was caused by elevated root lignification levels with activated expression of genes involved in the lignin biosynthesis pathway.

The AP2/ERF transcription factors were reported to be involved in lignin biosynthesis regulation by directly binding to DRE element or GCC-boxes in the promoters of the lignin biosynthetic genes. *OsERF71* was found to control lignin biosynthesis in roots by directly binding to the promoter of *OsCCR1*, which contains a GCC core cis-element (Lee et al., 2016). EjERF39 could transactivate the promoter of the lignin biosynthetic gene Ej4CL1 by recognizing the DRE element (Zhang et al., 2020). In the current study, an enhanced expression level of lignin biosynthetic genes was observed in OE lines, especially *CCR* and *COMT* (Figure 5C). The bioinformatic analysis of the *cis* elements in the promoter region of *CCR* revealed two DRE core elements to be present at the promoter of *CCR* (Supplementary Figure 2), suggesting the possibility that *IbRAP2.4* promoted lignin biosynthesis during SR formation by recognizing and directly binding to the DRE element within the promoter of *CCR*.

## Data Availability Statement

The datasets presented in this study can be found in online repositories. The names of the repository/repositories and accession number(s) can be found below: https://www.ncbi.nlm.nih.gov/, KX255653.1 and https://www.ncbi.nlm.nih.gov/, ARS72979.1.

## Author Contributions

XB and YY conceived and designed the research. XB, HK, QZ, SL, and PM performed the experiments. XB, S-SK, ZJ, and YX analyzed the data. PZ and YY interpreted the data. XB, PZ, and YY wrote the manuscript. All authors read and approved the manuscript.

## Conflict of Interest

The authors declare that the research was conducted in the absence of any commercial or financial relationships that could be construed as a potential conflict of interest.

## Publisher’s Note

All claims expressed in this article are solely those of the authors and do not necessarily represent those of their affiliated organizations, or those of the publisher, the editors and the reviewers. Any product that may be evaluated in this article, or claim that may be made by its manufacturer, is not guaranteed or endorsed by the publisher.

## Footnotes

1
http://www.ncbi.nlm.nih.gov/Structure/cdd/wrpsb.cgi

2
http://www.ncbi.nlm.nih.gov/

3
https://www.megasoftware.net/

4
http://en.bio-soft.net/format/BioEdit.html

5
http://public-genomes-ngs.molgen.mpg.de/cgi-bin/hgGateway?db=ipoBat4

6
http://bioinformatics.psb.ugent.be/webtools/plantcare/html/

7
https://www.genscript.com/tools/real-time-pcr-taqman-primer-design-tool

8
http://www.promega.com

## Supplementary Material

Supplementary Figure 1 Phylogenetic analysis of IbRAP2.4 homologs in plants.

Supplementary Figure 2 Characterization of *cis*-acting elements in the promoter regions of *IbRAP2.4* and *CCR*.

Supplementary Figure 3 Phenotypic and physiological characterization of *Arabidopsis* transgenic plants overexpressing *IbRAP2.4* (OV1 and OV2). Data represent mean ± SD of three biological replicates. Asterisks indicate significant differences between WT and OV plants at ***P* < 0.01.

Supplementary Figure 4 Phenotypic characterization of sweetpotato transgenic plants overexpressing *IbRAP2.4* (OE1, OE2, and OE3) in the field.

## References

[B1] Ahmed M. A. Zarebanadkouki M. Kaestner A. Carminati A. (2016). Measurements of water uptake of maize roots: the key function of lateral roots. *Plant Soil.* 398 59–77. 10.1007/s11104-015-2639-6

[B2] Belehu T. Hammes P. S. Robbertse P. J. (2004). The origin and structure of adventitious roots in sweet potato (*Ipomoea batatas*). *Aust. J. Bot.* 52:551. 10.1071/BT03152

[B3] Cai X.-T. Xu P. Zhao P.-X. Liu R. Yu L.-H. Xiang C.-B. (2014). Arabidopsis ERF109 mediates cross-talk between jasmonic acid and auxin biosynthesis during lateral root formation. *Nat. Commun.* 5:5833. 10.1038/ncomms6833 25524530

[B4] Clough S. J. Bent A. F. (1998). Floral dip: a simplified method for Agrobacterium-mediated transformation of *Arabidopsis thaliana*. *Plant J.* 16 735–743. 10.1046/j.1365-313x.1998.00343.x 10069079

[B5] Du Y. Scheres B. (2018). Lateral root formation and the multiple roles of auxin. *J. Exp. Bot.* 69 155–167. 10.1093/jxb/erx223 28992266

[B6] El Sheikha A. F. Ray R. C. (2017). Potential impacts of bioprocessing of sweet potato: review. *Crit. Rev. Food. Sci. Nutr.* 57 455–471. 10.1080/10408398.2014.960909 25975980

[B7] Firon N. LaBonte D. Villordon A. Kfir Y. Solis J. Lapis E. (2013). Transcriptional profiling of sweetpotato (*Ipomoea batatas*) roots indicates down-regulation of lignin biosynthesis and up-regulation of starch biosynthesis at an early stage of storage root formation. *BMC Genomics.* 14:460. 10.1186/1471-2164-14-460 23834507 PMC3716973

[B8] Guan L. Tayengwa R. Cheng Z. Peer W. A. Murphy A. S. Zhao M. (2019). Auxin regulates adventitious root formation in tomato cuttings. *BMC Plant Biol.* 19:435. 10.1186/s12870-019-2002-9 31638898 PMC6802334

[B9] He S. Wang H. Hao X. Wu Y. Bian X. Yin M. (2021). Dynamic network biomarker analysis discovers IbNAC083 in the initiation and regulation of sweet potato root tuberization. *Plant. J.* 108 793–813. 10.1111/tpj.15478 34460981

[B10] Hirota A. Kato T. Fukaki H. Aida M. Tasaka M. (2007). The Auxin-regulated AP2/EREBP gene PUCHI is required for morphogenesis in the early lateral root primordium of *Arabidopsis*. *Plant Cell.* 19 2156–2168. 10.1105/tpc.107.050674 17630277 PMC1955702

[B11] Iwata Y. Lee M.-H. Koizumi N. (2011). Analysis of a transcription factor using transient assay in *Arabidopsis protoplasts*. *Methods Mol. Biol.* 754 107–117. 10.1007/978-1-61779-154-3_6 21720949

[B12] Jeong J. S. Kim Y. S. Baek K. H. Jung H. Ha S.-H. Do Choi Y. (2010). Root-specific expression of OsNAC10 improves drought tolerance and grain yield in rice under field drought conditions. *Plant Physiol.* 153 185–197. 10.1104/pp.110.154773 20335401 PMC2862432

[B13] Jeong J. S. Kim Y. S. Redillas M. C. F. R. Jang G. Jung H. Bang S. W. (2013). OsNAC5 overexpression enlarges root diameter in rice plants leading to enhanced drought tolerance and increased grain yield in the field. *Plant Biotechnol. J.* 11 101–114. 10.1111/pbi.12011 23094910

[B14] Kim S. H. Ahn Y. O. Ahn M.-J. Lee H.-S. Kwak S.-S. (2012). Down-regulation of β-carotene hydroxylase increases β-carotene and total carotenoids enhancing salt stress tolerance in transgenic cultured cells of sweetpotato. *Phytochemistry* 74 69–78. 10.1016/j.phytochem.2011.11.003 22154923

[B15] Kim Y.-H. Jeong J. C. Park S. Lee H.-S. Kwak S.-S. (2012). Molecular characterization of two ethylene response factor genes in sweetpotato that respond to stress and activate the expression of defense genes in tobacco leaves. *J. Plant Physiol.* 169 1112–1120. 10.1016/j.jplph.2012.03.002 22459326

[B16] Kitomi Y. Ito H. Hobo T. Aya K. Kitano H. Inukai Y. (2011). The auxin responsive AP2/ERF transcription factor CROWN ROOTLESS5 is involved in crown root initiation in rice through the induction of OsRR1, a type-A response regulator of cytokinin signaling: CRL5 is involved in crown root initiation in rice. *Plant J.* 67 472–484. 10.1111/j.1365-313X.2011.04610.x 21481033

[B17] Lee D.-K. Jung H. Jang G. Jeong J. S. Kim Y. S. Ha S.-H. (2016). Overexpression of the OsERF71 transcription factor alters iice root structure and drought resistance. *Plant Physiol.* 172 575–588. 10.1104/pp.16.00379 27382137 PMC5074616

[B18] Lee S.-Y. Hwang E. Y. Seok H.-Y. Tarte V. N. Jeong M. S. Jang S. B. (2015). *Arabidopsis* AtERF71/HRE2 functions as transcriptional activator via cis-acting GCC box or DRE/CRT element and is involved in root development through regulation of root cell expansion. *Plant Cell Rep.* 34 223–231. 10.1007/s00299-014-1701-9 25344007

[B19] Li Y. Zhang H. Zhang Q. Liu Q. Zhai H. Zhao N. (2019). An AP2/ERF gene, IbRAP2-12, from sweetpotato is involved in salt and drought tolerance in transgenic *Arabidopsis*. *Plant Sci.* 281 19–30. 10.1016/j.plantsci.2019.01.009 30824052

[B20] Lin R.-C. Park H.-J. Wang H.-Y. (2008). Role of Arabidopsis RAP2.4 in regulating light- and ethylene-mediated developmental processes and drought stress tolerance. *Mol. Plant.* 1 42–57. 10.1093/mp/ssm004 20031913

[B21] Liu G. Gao S. Tian H. Wu W. Robert H. S. Ding Z. (2016). Local transcriptional control of YUCCA regulates auxin promoted root-growth inhibition in response to aluminium stress in *Arabidopsis*. *PLoS Genet.* 12:e1006360. 10.1371/journal.pgen.1006360 27716807 PMC5065128

[B22] Livak K. J. Schmittgen T. D. (2001). Analysis of relative gene expression data using RealTime Quantitative PCR and the 2-△△CT Method. *Methods* 25 402–408. 10.1006/meth.2001.1262 11846609

[B23] Lv B. Wei K. Hu K. Tian T. Zhang F. Yu Z. (2021). MPK14-mediated auxin signaling controls lateral root development via ERF13-regulated very-long-chain fatty acid biosynthesis. *Mol. Plant.* 14 285–297. 10.1016/j.molp.2020.11.011 33221411

[B24] Motte H. Vanneste S. Beeckman T. (2019). Molecular and environmental regulation of root development. *Annu. Rev. Plant Biol.* 70 465–488. 10.1146/annurev-arplant-050718-100423 30822115

[B25] Nakano T. Suzuki K. Fujimura T. Shinshi H. (2006). Genome-wide analysis of the ERF gene family in *Arabidopsis* and rice. *Plant Physiol.* 140 411–432. 10.1104/pp.105.073783 16407444 PMC1361313

[B26] Noh S. A. Lee H.-S. Huh E. J. Huh G. H. Paek K.-H. Shin J. S. (2010). SRD1 is involved in the auxin-mediated initial thickening growth of storage root by enhancing proliferation of metaxylem and cambium cells in sweetpotato (*Ipomoea batatas*). *J. Exp. Bot.* 61 1337–1349. 10.1093/jxb/erp399 20150515 PMC2837253

[B27] Noh S. A. Lee H.-S. Kim Y.-S. Paek K.-H. Shin J. S. Bae J. M. (2013). Down-regulation of the IbEXP1 gene enhanced storage root development in sweetpotato. *J. Exp. Bot.* 64 129–142. 10.1093/jxb/ers236 22945944 PMC3528024

[B28] Phukan U. J. Jeena G. S. Tripathi V. Shukla R. K. (2017). Regulation of Apetala2/Ethylene response factors in plants. *Front. Plant Sci.* 8:150. 10.3389/fpls.2017.00150 28270817 PMC5318435

[B29] Placido D. F. Sandhu J. Sato S. J. Nersesian N. Quach T. Clemente T. E. (2020). The LATERAL ROOT DENSITY gene regulates root growth during water stress in wheat. *Plant Biotechnol. J.* 18 1955–1968. 10.1111/pbi.13355 32031318 PMC7415784

[B30] Qin H. Huang R. (2018). Auxin controlled by ethylene steers root development. *Int. J. Mol. Sci.* 19:3656. 10.3390/ijms19113656 30463285 PMC6274790

[B31] Redillas M. C. F. R. Jeong J. S. Kim Y. S. Jung H. Bang S. W. Choi Y. D. (2012). The overexpression of OsNAC9 alters the root architecture of rice plants enhancing drought resistance and grain yield under field conditions. *Plant Biotechnol. J.* 10 792–805. 10.1111/j.1467-7652.2012.00697.x 22551450

[B32] Uga Y. Sugimoto K. Ogawa S. Rane J. Ishitani M. Hara N. (2013). Control of root system architecture by DEEPER ROOTING 1 increases rice yield under drought conditions. *Nat. Genet.* 45 1097–1102. 10.1038/ng.2725 23913002

[B33] Waadt R. Kudla J. (2008). In planta visualization of protein interactions using bimolecular fluorescence complementation (BiFC). *CSH Protoc.* 2008:rot4995. 10.1101/pdb.prot4995 21356813

[B34] Wei K. Ruan L. Wang L. Cheng H. (2019). Auxin-induced adventitious root formation in nodal cuttings of *Camellia sinensis*. *Int. J. Mol. Sci.* 20:4817. 10.3390/ijms20194817 31569758 PMC6801801

[B35] Wilson L. A. Lowe S. B. (1973). The anatomy of the root system in west indian sweet potato (*Ipomoea batatas* (L.) Lam.) cultivars. *Ann. Bot.* 37 633–643. 10.1093/oxfordjournals.aob.a084729

[B36] Xiao G. Q. Qin H. Zhou J. H. Quan R. D. Lu X. Y. Huang R. F. (2016). OsERF2 controls rice root growth and hormone responses through tuning expression of key genes involved in hormone signaling and sucrose metabolism. *Plant Mol. Biol.* 90 293–302. 10.1007/s11103-015-0416-9 26659593 PMC4717165

[B37] Xie Z. Nolan T. M. Jiang H. Yin Y. (2019). AP2/ERF transcription factor regulatory networks in hormone and abiotic stress responses in *Arabidopsis*. *Front. Plant Sci.* 10:228. 10.3389/fpls.2019.00228 30873200 PMC6403161

[B38] Yang S. U. Kim H. Kim R. J. Kim J. Suh M. C. (2020). AP2/DREB transcription factor RAP2.4 activates cuticular wax biosynthesis in *Arabidopsis* leaves under drought. *Front. Plant Sci.* 11:895. 10.3389/fpls.2020.00895 32719695 PMC7347990

[B39] Yoon J. Cho L.-H. Yang W. Pasriga R. Wu Y. Hong W.-J. (2020). Homeobox transcription factor OsZHD2 promotes root meristem activity in rice by inducing ethylene biosynthesis. *J. Exp. Bot.* 71 5348–5364. 10.1093/jxb/eraa209 32449922 PMC7501826

[B40] Yu Y. Kim H. S. Ma P. Jia Z. Guo X. Xie Y. (2020). A novel ethylene-responsive factor IbERF4 from sweetpotato negatively regulates abiotic stress. *Plant Biotechnol. Rep.* 14 397–406. 10.1007/s11816-020-00612-x

[B41] Zhang J. Yin X.-R. Li H. Xu M. Zhang M.-X. Li S.-J. (2020). Ethylene response FACTOR39-MYB8 complex regulates low-temperature-induced lignification of loquat fruit. *J. Exp. Bot.* 71 3172–3184. 10.1093/jxb/eraa085 32072171 PMC7475177

[B42] Zhang J.-Y. Broeckling C. D. Blancaflor E. B. Sledge M. K. Sumner L. W. Wang Z.-Y. (2005). Overexpression of WXP1, a putative *Medicago truncatula* AP2 domain-containing transcription factor gene, increases cuticular wax accumulation and enhances drought tolerance in transgenic alfalfa (*Medicago sativa*). *Plant J.* 42 689–707. 10.1111/j.1365-313X.2005.02405.x 15918883

[B43] Zhang J.-Y. Broeckling C. D. Sumner L. W. Wang Z.-Y. (2007). Heterologous expression of two *Medicago truncatula* putative ERF transcription factor genes, WXP1 and WXP2, in Arabidopsis led to increased leaf wax accumulation and improved drought tolerance, but differential response in freezing tolerance. *Plant Mol. Biol.* 64 265–278. 10.1007/s11103-007-9150-2 17347795

[B44] Zhao Y. Cheng S. Song Y. Huang Y. Zhou S. Liu X. (2015). The interaction between rice ERF3 and WOX11 promotes crown root development by regulating gene expression involved in cytokinin signaling. *Plant Cell.* 27 2469–2483. 10.1105/tpc.15.00227 26307379 PMC4815106

