# Supplementary figures and images for: Different Functions of IbRAP2.4, a Drought-Responsive AP2/ERF Transcription Factor, in Regulating Root Development Between Arabidopsis and Sweetpotato
Source: Front Plant Sci. 2022 Jan 26;13:820450. doi: 10.3389/fpls.2022.820450 (PMC8826056; doi:10.3389/fpls.2022.820450)

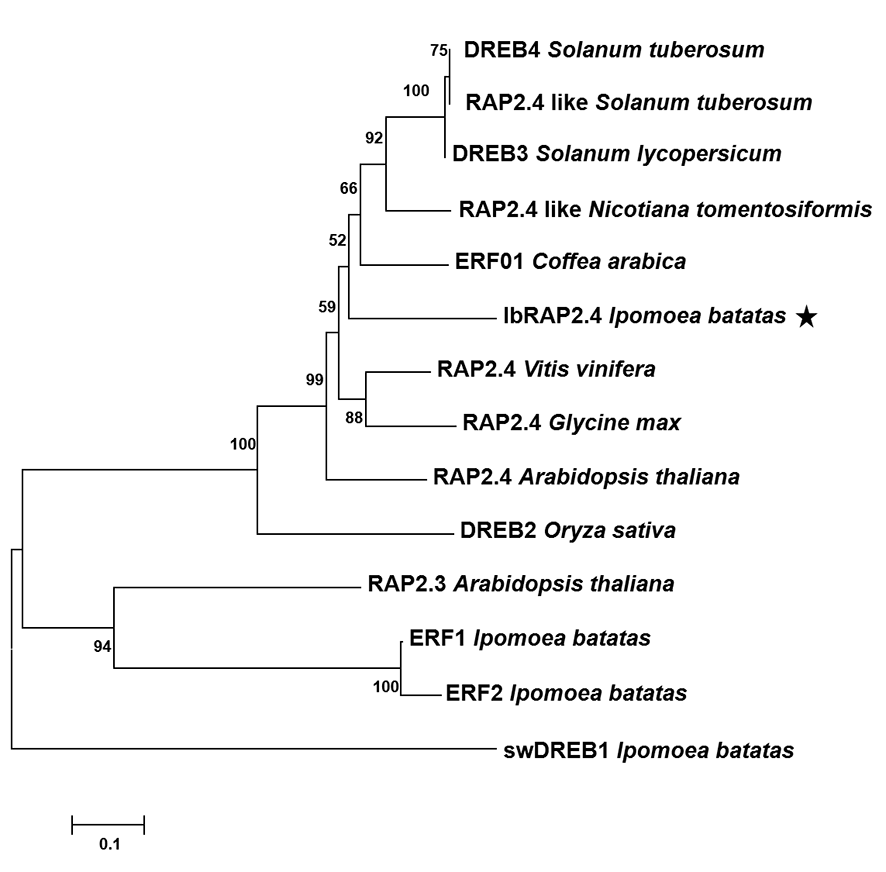

Supplement: Supplementary Figure 1 — Phylogenetic analysis of IbRAP2.4 homologs in plants. [file Image_1.TIF]

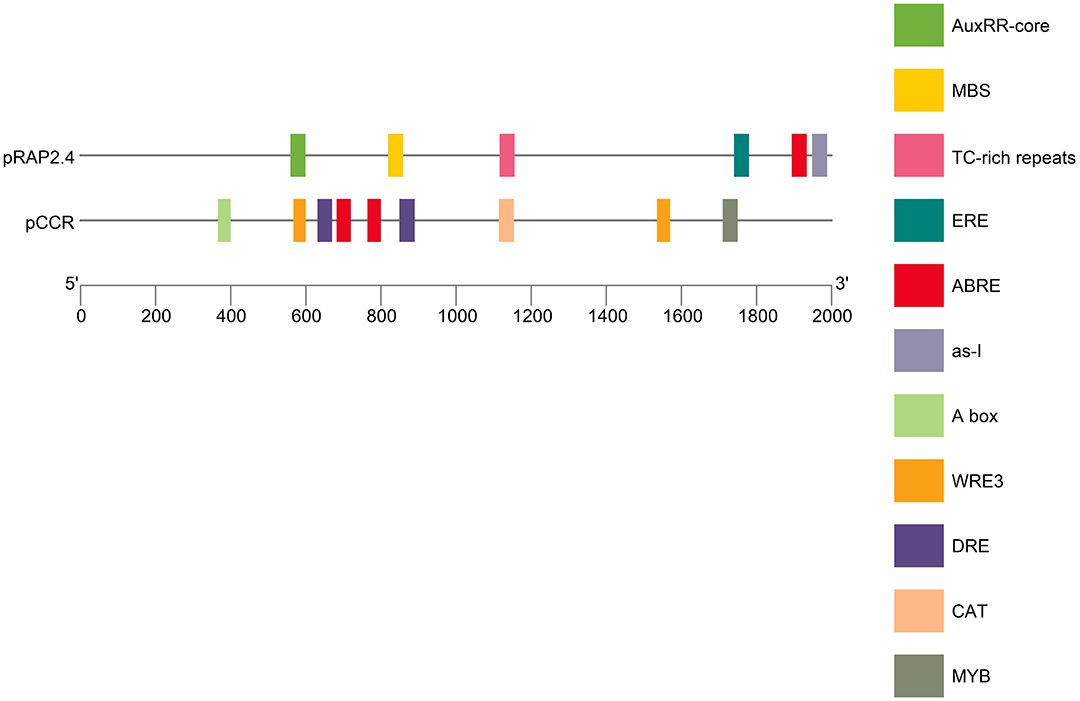

Supplement: Supplementary Figure 2 — Characterization of cis-acting elements in the promoter regions of IbRAP2.4 and CCR. [file Image_2.JPEG]

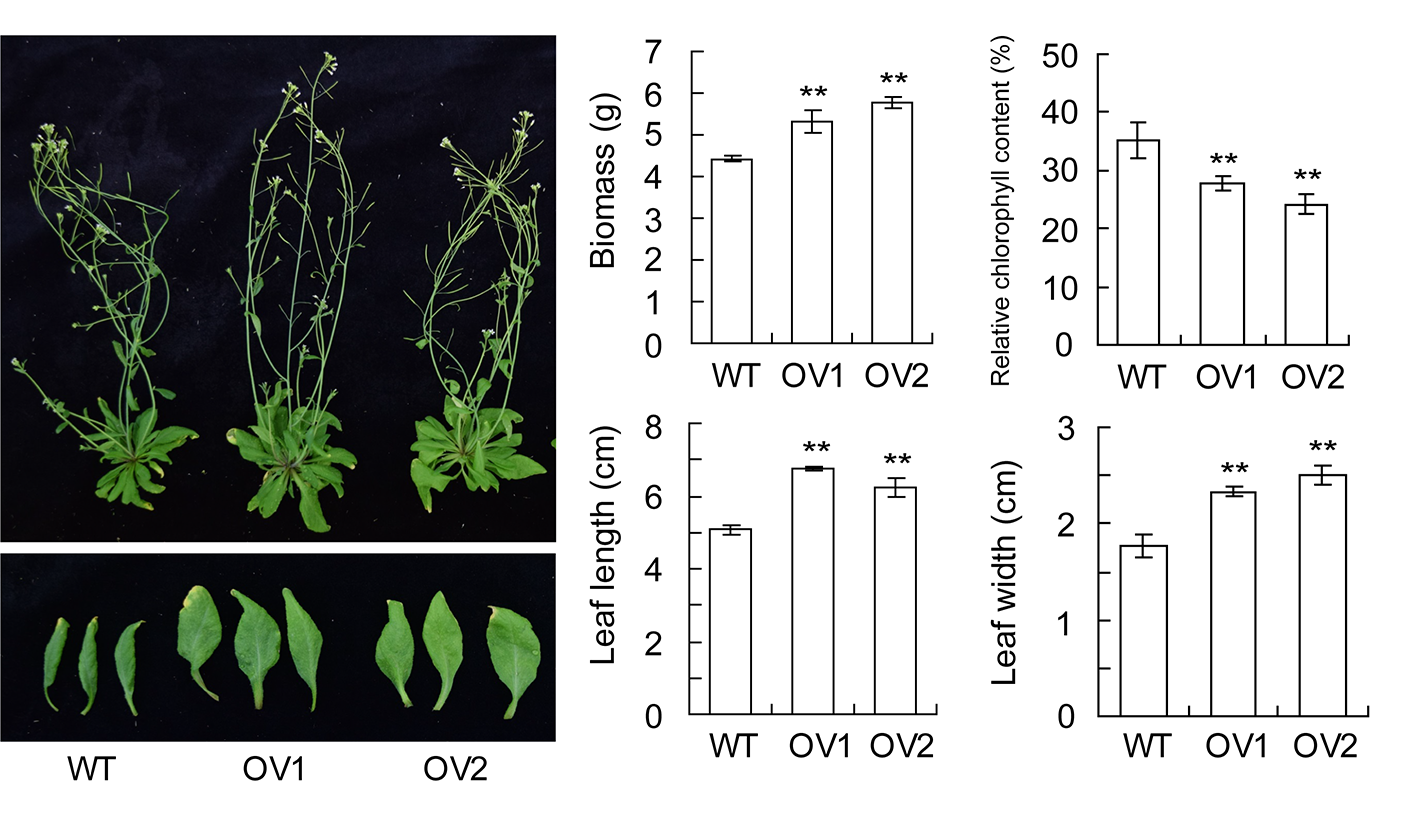

Supplement: Supplementary Figure 3 — Phenotypic and physiological characterization of Arabidopsis transgenic plants overexpressing IbRAP2.4 (OV1 and OV2). Data represent mean ± SD of three biological replicates. Asterisks indicate significant differences between WT and OV plants at **P < 0.01. [file Image_3.TIF]

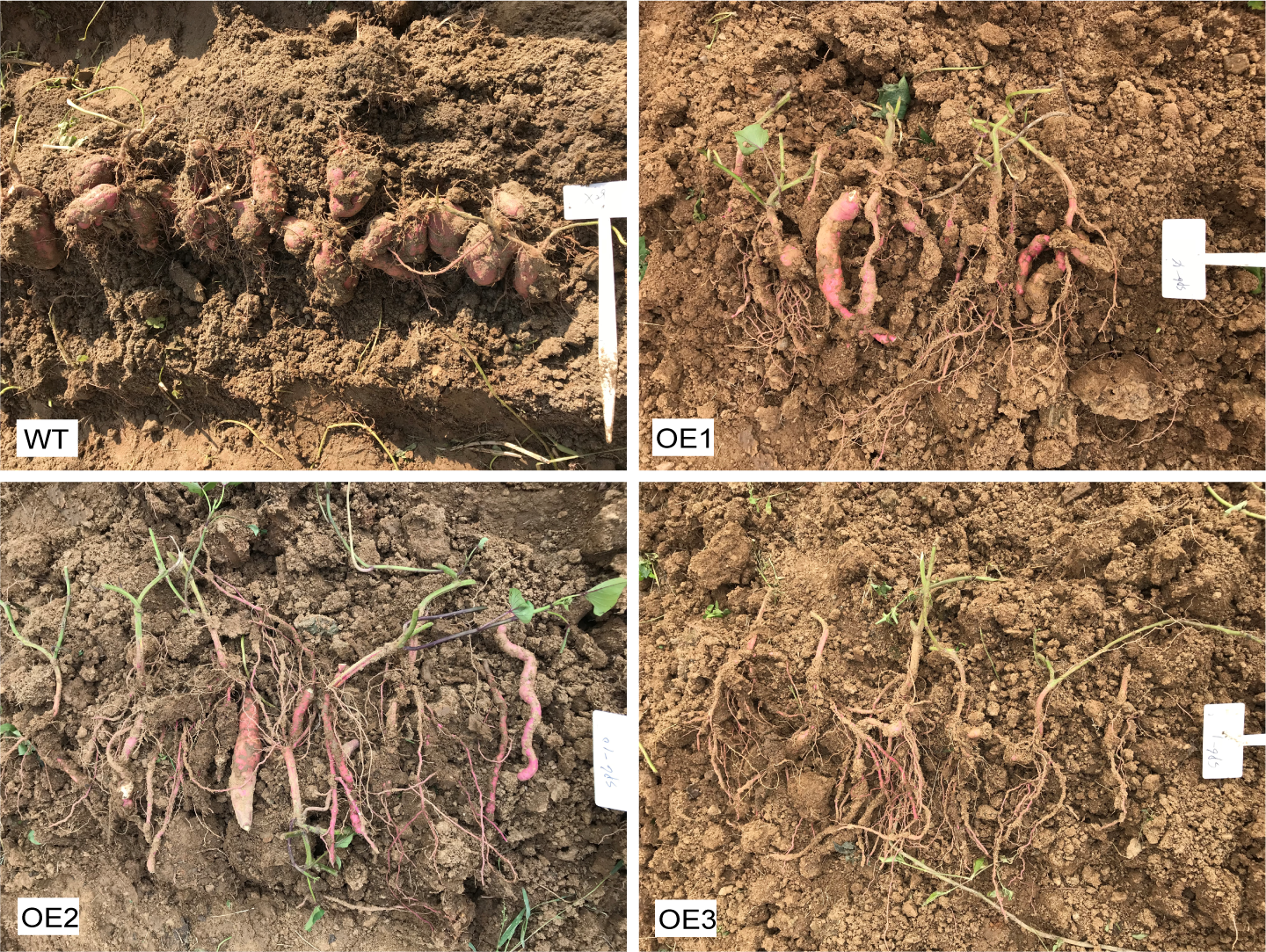

Supplement: Supplementary Figure 4 — Phenotypic characterization of sweetpotato transgenic plants overexpressing IbRAP2.4 (OE1, OE2, and OE3) in the field. [file Image_4.TIF]
