# Supplementary material for: Different Functions of IbRAP2.4, a Drought-Responsive AP2/ERF Transcription Factor, in Regulating Root Development Between Arabidopsis and Sweetpotato
Source: Front Plant Sci. 2022 Jan 26;13:820450. doi: 10.3389/fpls.2022.820450 (PMC8826056; doi:10.3389/fpls.2022.820450)
Supplement: Supplementary file 5 [file Table_1.DOC]

Supplement Table S1 Primer pairs sequence.

| Marker | | Forward sequence (5’-3’) | | Reverse sequence (5’-3’) |
| --- | --- | --- | --- | --- |
| Cloning | | | | |
| RAPC | CACGAGGGCTTATTAGTTGTCC | | CCCACCCTACACCAATCATCAC | |
| Vector construction Primers | | | | |
| RAPY | | GGCCAGGCCTCCATGGATGGCTGCCACTATTGATAT | | CCAAGATATACCATGGTGACCCGCCCGAACCTGAAT |
| RAPG | | AGGTACCCGGGGATCCATGGCTGCCACTATTGATAT | | GCAGGTCGACTCTAGATGACCCGCCCGAACCTGAAT |
| ORAP | | GACTCTAGAAAGCTTATGGCTGCCACTATTGATAT | | TTCGAGCTCGGTACCTGACCCGCCCGAACCTGAAT |
| PCR identification of transgenic plants | | | | |
| *Hyg* | | ACACAGCCATCGGTCCAGAC | | ATCTTAGCCAGACGAGCGGG |
| Quantitative PCR Primers | | | | |
| RTTublin | CAACTACCAGCCACCAACTGT | | CAAGATCCTCACGAGCTTCAC | |
| RTRAP | CGTCACCGTCGCCTTCTTTGT | | GCGTGGAGCCCATCATTTGTT | |
| RTPAL | GGCGAGCACGAGAAGAATGT | | ATGGCAGGGTTTCCGTTCTC | |
| RTC4H | GCTTCAAGTCGGCGATGATT | | GGCGAGGATACGACCACTAA | |
| RTCAD | AGCTGGTAATGGTTGGCATC | | TCCAAAGCCGTGTTGACATA | |
| RTCCR | GCAGAGATAACGGCCAGAAG | | TTGCTACAACCCACCATCAA | |
| RTCOMT | AAACGGGAAAGTGATCGTTG | | CCATGATCCAAGTGTTGACG | |
| RTCCoAOMT | CCGGTTCTTGACCAGATGAT | | TTCCACAGGGTGTTGTCGTA | |
